# Docosahexaenoic Acid-Enhanced Autophagic Flux Improves Cardiac Dysfunction after Myocardial Infarction by Targeting the AMPK/mTOR Signaling Pathway

**DOI:** 10.1155/2022/1509421

**Published:** 2022-02-27

**Authors:** Youyang Shi, Hao Li, Tingting Wu, Qiaoyu Wang, Qiongjun Zhu, Xueqiang Guan, Rongzhou Wu

**Affiliations:** ^1^Children's Heart Center, The Second Affiliated Hospital and Yuying Children's Hospital of Wenzhou Medical University, Wenzhou, Zhejiang 325000, China; ^2^Department of Cardiology, The Second Affiliated Hospital and Yuying Children's Hospital of Wenzhou Medical University, Wenzhou, Zhejiang 325000, China

## Abstract

*Background and Purpose*. Docosahexaenoic acid (DHA) is a type of polyunsaturated fatty acid enriched in cod liver oil and seaweed. It is necessary for the human body and has important functions, such as antioxidation and antiatherosclerosis activities. Long-term oral administration of DHA or the use of DHA at the initial stage of ischemia can increase the level of autophagy and exert a protective effect on neurological functions related to cerebral infarction. However, the effect of DHA on myocardial injury and cardiac insufficiency after myocardial infarction (MI) is unknown. This study was aimed at exploring whether DHA plays a protective role in AMI and its specific molecular mechanism. *Experimental Method*. In vitro cardiomyocyte hypoxia and in vivo MI injury models were used to determine the role of DHA in MI. Hypoxic injury induced damage in cultured neonatal mouse cardiomyocytes (NMCs). The C57BL/6J mouse MI model was established by permanent ligation of the left anterior descending branch. *Main Results*. DHA improved the cardiomyocyte viability of NMCs induced by hypoxia injury and reduced cell necrosis. DHA reduced infarct size, improved heart function, and reduced the degree of myocardial fibrosis in mice after MI. In addition, DHA enhanced autophagy flux and reduced apoptosis in vitro and in vivo. In addition, we found that chloroquine, an autophagy inhibitor, blocked the protective effect of DHA on cardiomyocyte apoptosis and cardiac dysfunction, indicating that DHA exerts cardioprotective effects in part by promoting autophagy flux. We also observed that DHA enhanced autophagy flux by activating the AMPK/mTOR signaling pathway. *Conclusions and Significance*. In conclusion, our findings indicate for the first time that DHA improves MI-induced cardiac dysfunction by promoting AMPK/mTOR-mediated autophagic flux.

## 1. Introduction

Acute myocardial infarction (AMI) refers to myocardial tissue necrosis due to acute and persistent ischemia, as well as hypoxia in the coronary arteries [1, 2]. With the improvement of the global economy, accelerated pace of life, and combined effects of various social factors, the morbidity and mortality rates of AMI have been increasing worldwide. AMI causes irreversible death of cardiomyocytes, which can lead to pathological myocardial remodeling, including progressive ventricular dilation and impaired ventricular pumping function and ultimately result in heart failure [3]. Although the coronary arteries can be recanalized through percutaneous coronary angioplasty, thrombolysis, laser therapy, and other traditional coronary artery recanalization therapies, cardiomyocyte death still occurs due to ischemia and hypoxia. Therefore, to reduce post-MI ventricular remodeling and cardiac dysfunction, there is an urgent need for more effective drugs and more therapeutic targets [4].

Studying cardiomyocyte apoptosis and autophagy during the pathophysiological process of poor cardiac remodeling post-MI may reveal effective therapeutic targets [5]. Much evidence indicates that cardiomyocytes initiate apoptosis in the initial stage of ischemia [6]. Macroautophagy (referred to as autophagy from here on) first synthesizes double-membrane cytoplasmic vesicles and then wraps the cellular components in damaged cells to form autophagosomes and combines them with lysosomes to form autolysosomes. Finally, macromolecular substances are degraded in the cells to produce amino acids and energy, which are then recycled. This dynamic process is referred to as autophagy flux.

Under normal conditions, autophagy occurs at low basal levels, but under stress—such as hypoxia, insufficient energy supply, or pathogen invasion—autophagy is overactivated [7, 8]. An AMI mouse-model study showed that myocardial ischemia significantly induced autophagy in surviving cardiomyocytes; continued use of autophagy agonists partially recovered cardiac function and promoted ventricular remodeling [9]. These data imply that autophagy may play an important role in AMI by affecting the survival of the hibernating myocardium.

Docosahexaenoic acid (DHA, C_22_H_32_O_2_) is produced by desaturation and elongation reactions of alpha linolenic acid. DHA, eicosapentaenoic acid (another product of alpha linolenic acid), and the precursor alpha linolenic acid are omega-3 polyunsaturated fatty acids, which are essential fatty acids for the human body. Previous research demonstrated that long-term oral administration of DHA or use of DHA at the initial stage of ischemia can significantly induce autophagy, limit the size of cerebral infarction, and exert neuroprotective effects [10–13]. Therefore, DHA-induced autophagy may play a role in reducing myocardial injury and improving cardiac function post-MI.

Adenosine monophosphate-activated protein kinase (AMPK) is a classic signaling protein that induces autophagy [14]. Mammalian target of rapamycin (mTOR) is the downstream signaling molecule of AMPK, which also participates in the regulation of autophagy [15]. Previous studies indicate that DHA can activate AMPK in a variety of disease models [16, 17]. This study explored the ability of DHA to enhance autophagic flux through the AMPK/mTOR signaling pathway in an AMI mouse model.

## 2. Materials and Methods

### 2.1. Animal Experiments and Mouse Model of MI

The animal experiments involved in this study complied with the Guidelines for the Care and Use of Laboratory Animals formulated by the Ministry of Science and Technology of China and were approved by the Ethics Committee of Wenzhou Medical University (No. SYXK [Zhejiang] 2015-0009). C57BL/6J male mice (aged 6–8 weeks) weighing 20–25 g were obtained from the Beijing Weitong Lihua Experimental Animal Center and kept in an environment with constant temperature and humidity at the Experimental Animal Center of Wenzhou Medical University. MI was induced in the mice as previously described [18]. Briefly, the mice were anesthetized with 2% isoflurane/oxygen inhalation anesthesia and MI was induced by permanent ligation of the left coronary artery with seven to ten sutures. After ligation, the anterior wall of the left ventricle became grey in color due to ischemia, and heart pulsation weakened, indicating that the LAD ligation was successful. The sham-operated mice underwent the same procedure without ligation. After closing the chest cavity, the mice were placed on a constant temperature pad, and the model was successfully established after resuscitation. The mice that died within 2–3 hours after surgery were excluded from further analysis. Mice were sacrificed at 24 hours or 28 days after MI.

**Figure 1:**
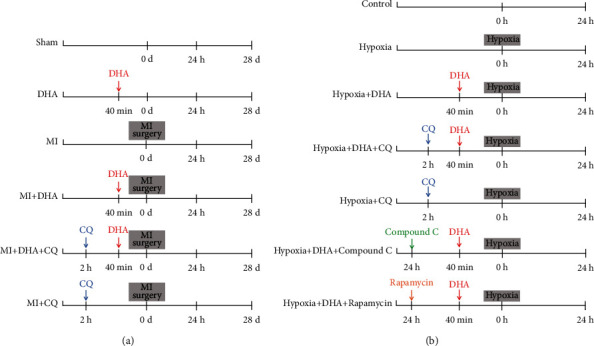
(a) Protocol of the in vivo study. (b) Protocol of the in vitro study.

The mice were randomly divided into the following six groups: sham operation group administered phosphate-buffered saline (PBS) (intraperitoneal, i.p.); DHA group, given DHA (375 mg/kg body weight, i.p.; Sigma-Aldrich, St. Louis, MO, USA); MI group, administered the same amount of PBS (i.p.); MI+DHA group, given DHA (375 mg/kg body weight, i.p.); MI+DHA+chloroquine (CQ) group, administered DHA (375 mg/kg body weight, i.p.) and CQ (10 mg/kg body weight, i.p.; Sigma); and MI+CQ group given PBS and CQ (10 mg/kg body weight, i.p.). The DHA was administered at an appropriate dose (375 mg/kg body weight) for rats or mice through i.p. injection, as described elsewhere [19]. Each group of mice was checked daily for 4 weeks after coronary artery surgery. The animals were sacrificed at designated time points and heart tissues were collected for further analysis. The in vivo experimental protocol is shown in Figure 1(a).

### 2.2. Cardiomyocyte Isolation and Culture

Neonatal mouse cardiomyocytes (NMCs) were isolated from the heart ventricles of 1–2-day-old C57BL/6J mice using a previously described method [20]. NMCs were cultured in an incubator at 37°C (gas mixture: 95% O_2_ and 5% CO_2_). The cells were initially cultured in Dulbecco's modified Eagle's medium (DMEM) containing 10% HS for 72 hours. They were then randomly divided into the following groups: control group; hypoxia group, in which myocardial cells were cultured under hypoxic conditions (37°C, 1% O_2_, 5% CO_2_, and 94% N_2_ gas mixture) for 24 hours to stimulate cardiomyocyte damage; hypoxia+DHA group, involving “hypoxia culture” with DHA (1,500 *μ*M); hypoxia+DHA+CQ group, involving hypoxia culture with DHA (1,500 *μ*M) and CQ (30 *μ*M); hypoxia+CQ group, involving hypoxia culture with CQ (30 *μ*M); hypoxia+DHA+compound C group, involving hypoxia culture with DHA (1,500 *μ*M) and compound C (10 *μ*M); and hypoxia+DHA+rapamycin group, involving hypoxia culture with DHA (1,500 *μ*M) and rapamycin (200 *μ*M). After 24 hours of incubation, the NMCs were harvested and prepared for analysis. The in vitro experimental protocol is shown in Figure 1(b).

### 2.3. Cell Viability Assay

A Cell Counting Kit-8 (CCK-8; Dojindo Co., Kumamoto, Japan) was used to measure cell viability according to the manufacturer's instructions. Primary mouse cells were inoculated on a 96-well plate (10^4^ cells/well) and cultured in maintenance medium at 37°C for 24 hours. The cells were treated with different concentrations of DHA (0.5, 1, 1.5, 2, 2.5, or 3 mM) and DMEM, as described above. The cells were then cultured under hypoxia for 24 hours, while the control group was cultured in a normoxic incubator at 37°C. After the treatment, the cells were washed with PBS before 100 *μ*L of nonfetal bovine serum (FBS) DMEM medium containing 10 *μ*L of CCK-8 solution was added to each well. The plates were incubated for another 2 hours and a microplate reader was used to measure the absorbance at 450 nm. Each group was tested using three replicate wells. The cell survival and proliferation rates of the treated cells were calculated relative to the control group.

### 2.4. Western Blotting

Mouse hearts and harvested NMCs were lysed in RIPA buffer and protein concentrations were measured using a BCA protein assay kit (Thermo Scientific, Waltham, MA, USA). The proteins were separated by SDS-PAGE, transferred to a polyvinylidene fluoride (PVDF) membrane, and incubated in 5% skim milk blocking buffer at room temperature for 2 hours. Finally, the membranes were incubated with the antibody and scanned using a chemiluminescence system (Amersham Bioscience, Amersham, UK). The primary antibodies used in this study were anti-LC3 B (Cat #14600-1-AP; Proteintech group, Rosemont, IL, USA), anti-p62 (Cat #23214; CST, Danvers, MA, USA), anti-caspase-3 (Cat #9662S; CST), antiphosphorylated AMPK (p-AMPK; Cat #2535S; CST), anti-AMPK (Cat #2532S; CST), antiphosphorylated mTOR (p-mTOR; Cat #5536S; CST), anti-mTOR (Cat #2972S; CST), and anti-GAPDH (1 : 10,000, Cat #AP0063; Boster, Shanghai, China). A 1 : 5,000-diluted secondary antibody was also used (Boster).

### 2.5. Triphenyltetrazolium Chloride (TTC) Staining for Assessment of Myocardial Infarct Size

At 24 hours after myocardial infarction (MI), mouse hearts were quickly frozen at −80°C and cut into 1-mm-thick sections. The sections were stained with 2% TTC for 30 minutes at room temperature. Viable tissue was stained red by the TTC, whereas the infarcted myocardium was not stained by the dye and appeared whiter than other areas. The infarct area size ratio was calculated using Image-Pro Plus software (Media Cybernetics, Rockville, IL, USA) as the ratio of infarct area to heart area.

### 2.6. Transthoracic Echocardiography

The mice were sedated with 2% isoflurane inhalation at 1 and 4 weeks after MI, and echocardiographs were recorded using a Vevo 1100 system equipped with a 30-MHz transducer (Visual Sonics, Canada). LV internal diameter at diastole (LVIDd) and LV internal diameter at systole (LVIDs) were measured based on an M-mode echocardiogram of the short axis of the left ventricle. The LV ejection fraction (LVEF) and LV fraction shortening (LVFS) values were calculated using computer algorithms.

### 2.7. Cardiac Histology and Immunochemistry

Heart tissues were fixed with 4% paraformaldehyde, dehydrated stepwise with sucrose, and embedded in oxytetracycline (OTC). The samples were then cut into 5 *μ*m slices on a microtome and subjected to Masson's trichrome staining (Solarbio, Beijing, China) and immunohistochemical staining. Masson's three-color staining method dyes collagen in the fibrotic infarct area blue. The stained sections were analysed under an optical microscope.

### 2.8. Apoptosis Assay (Terminal Deoxynucleotidyl Transferase dUTP Nick End Labeling [TUNEL] Staining)

A TUNEL detection kit (Roche, Mannheim, Germany) was used according to the manufacturer's protocol to analyze cell apoptosis; the results were observed under a fluorescence microscope. The nuclei of TUNEL-positive cells were stained green in the fluorescence images. The percentage of TUNEL-stained cells was calculated based on the distribution of cardiomyocyte staining in the captured images.

### 2.9. Immunofluorescence Evaluation of Fluorescent LC3 and p62 Puncta

NMCs were seeded in a confocal Petri dish and randomly assigned to the abovementioned groups. The cells were fixed with 4% paraformaldehyde at room temperature for 30 minutes. After washing with PBS, the cells were permeabilized with 0.2% Triton X-100. After blocking with 5% BSA, the cells were incubated with specific primary antibodies (anti-p62 or anti-LC3B,1 : 200) at 4°C overnight. Cells were washed three times the next day, incubated with secondary antibodies (red antirabbit fluorescent secondary antibody combined with LC3 and green antimouse fluorescent secondary antibody combined with p62) in a dark room at 37°C for 30 minutes, and labeled with DAPI. The remaining dye solution was washed away and imaging was performed using a confocal microscope.

### 2.10. Statistical Analysis

Results are presented as means ± SEM. Statistically significant differences were evaluated using one-way analysis of variance followed by Tukey's test for multiple comparisons. The analyses were performed using Prism (v. 7; GraphPad Software Inc., San Diego, CA, USA). The differences were considered statistically significant at *P* < 0.05.

## 3. Results

### 3.1. DHA Improved Cardiomyocyte Viability and Cardiac Dysfunction

**Figure 2:**
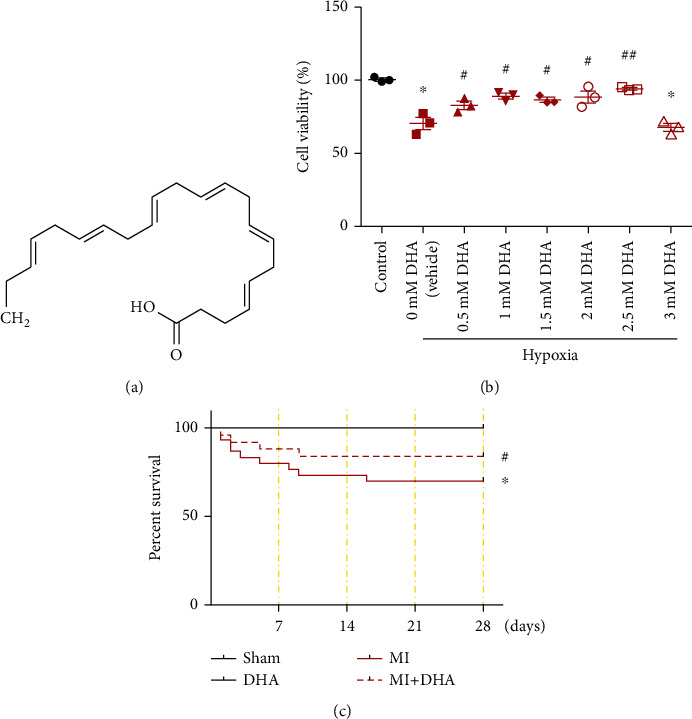
DHA improved cardiomyocyte viability and mouse survival. (a) The chemical structure of DHA. (b) Cytotoxic effects of DHA on hypoxia-treated NMCs by CCK8 assay. Data are means ± SEM. ^∗^*P* < 0.05 vs. control, ^#^*P* < 0.05 vs. vehicle, *n* = 3. (c) Survival curve of MI mice treated with DHA. Data are means ± SEM. ^∗^*P* < 0.05 vs. sham, ^#^*P* < 0.05 vs. MI, *n* = 6.

The chemical structure of DHA is shown in Figure 2(a). To determine the role of DHA in cardiomyocyte injury, various concentrations (500, 1,000, 1,500, 2,000, 2,500, and 3,000 *μ*M) of DHA were exposed to hypoxia-induced NMCs. CCK-8 analysis indicated that hypoxia led to cell death, while DHA treatment reversed NMC death and significantly enhanced cell viability. NMCs treated with 1,500 *μ*M DHA showed the highest level of cell viability (Figure 2(b)). Therefore, 1,500 *μ*M DHA was chosen for the in vitro experiments in this study. The role of DHA in post-AMI mice was examined at 4 weeks after MI. Survival analysis indicated that 21 of 30 mice in the MI group survived, whereas 26 of 30 mice survived in the MI+DHA group. No mice died in the DHA or sham group (Figure 2(c)).

**Figure 3:**
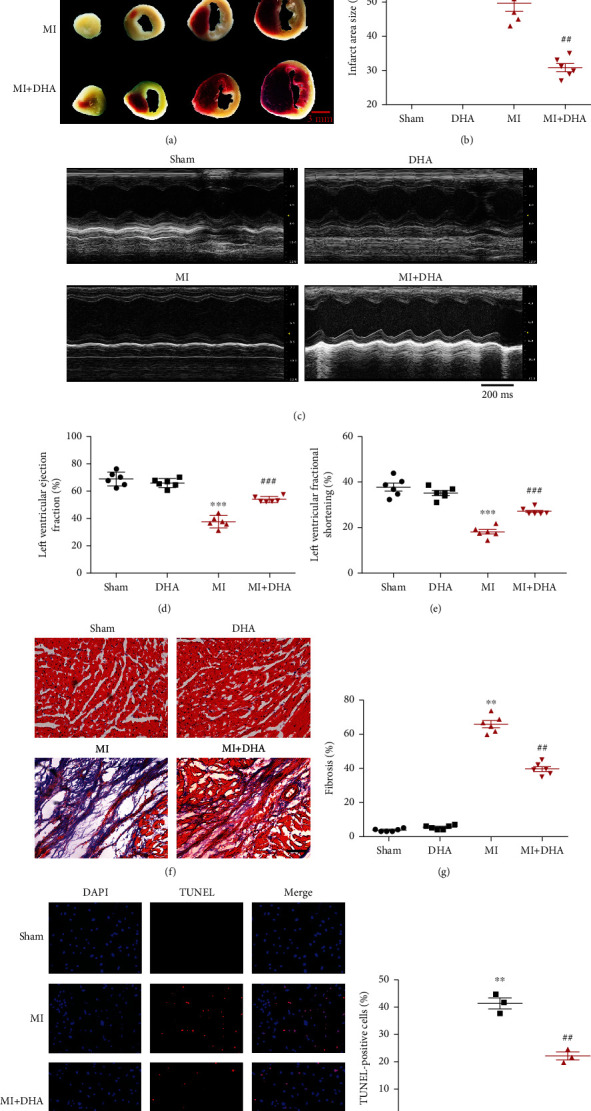
DHA improved MI-induced cardiac dysfunction and inhibited cardiac apoptosis. (a and b) TTC staining was performed to assess the infarct area of MI mice and calculate the infarct area size ratio (infarct area size ratio = infarct area ÷ total area of the heart; scale bar 3 mm). Data are means ± SEM. ^∗∗^*P* < 0.01 vs. sham, ^##^*P* < 0.01 vs. MI, *n* = 6. (c–e) Cardiac function analysed by echocardiography. (f and g) Myocardial fibrosis assessed by Masson's staining (original magnification ×200; scale bar 50 *μ*m). Data are means ± SEM. ^∗∗∗^*P* < 0.001 vs. sham, ^###^*P* < 0.001 vs. MI, *n* = 6. (h) Apoptosis of NMCs by TUNEL staining (red; original magnification ×200; scale bar 50 *μ*m). (i) Quantitative analyses of TUNEL-positive cells. Data are means ± SEM. ^∗∗^*P* < 0.01 vs. control, ^##^*P* < 0.01 vs. hypoxia, *n* = 3.

TTC staining was used to assess the extent of MI at 24 hours after AMI. No white areas were found in the myocardial tissues of the sham or DHA group, indicating no MI. The MI and MI+DHA groups showed obvious white infarct areas, implying that the mouse AMI model was successful. The white infarct area was significantly smaller in the MI+DHA group than in the MI group. This implies that DHA pretreatment reduced the area of MI post-AMI (Figures 3(a) and 3(b)). Masson's staining also showed that DHA treatment significantly reduced myocardial fibrosis in post-AMI mice (Figures 3(f) and 3(g)).

Echocardiography was used to assess the cardiac function and structure of mice at 4 weeks after coronary ligation. Echocardiographic analysis showed that the LVEF and LVFS values of the MI group were significantly lower than those of the sham group and improved with DHA treatment (Figures 3(c)–3(e)). Compared to the sham group, LVIDd and LVIDs values were significantly higher in the MI group, but after DHA treatment left ventricular enlargement was decreased (Figure S1).

### 3.2. DHA Inhibited Cardiac Apoptosis

Previous studies confirmed that apoptosis is an important cause of myocardial cell death after MI [6]. To determine whether DHA can protect the myocardium from apoptosis after infarction in vivo and in vitro, TUNEL staining and western blot analysis were used to evaluate the expression of apoptosis biomarkers. The results of in vitro TUNEL staining showed that DHA significantly inhibited the apoptosis of hypoxia-induced NMC (Figures 3(h) and 3(i)).

### 3.3. DHA-Enhanced Autophagic Flux in Hypoxia-Treated NMCs

DHA induces autophagy in several disease models [21]. To determine whether DHA treatment can promote the autophagy flux seen in hypoxia-induced NMC, the protein expression of the autophagy-associated molecules LC3 II/GAPDH and p62 was evaluated after DHA administration. LC3 is a marker protein of autophagy that participates in the formation of autophagosomes. LC3 II/GAPDH are generally used to assess the number of autophagosomes. SQSTM1/p62 can interact with ubiquitin and induce proteasomes or lysosomes to degrade proteins, which can be used as a reliable indicator of autophagy degradation [22].

**Figure 4:**
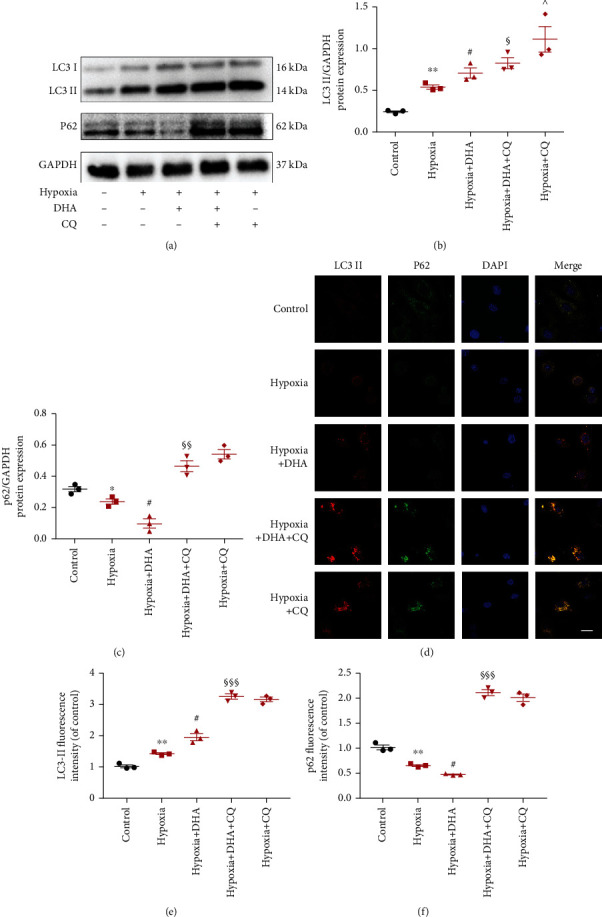
DHA-enhanced autophagic flux in hypoxia-treated NMCs. (a) LC3-II, p62, and GAPDH protein levels in NMCs by western blotting. (b and c) Quantification of LC3-II/GAPDH and P62/GAPDH. Data are means ± SEM. ^∗^*P* < 0.05 and ^∗∗^*P* < 0.01 vs. control, ^#^*P* < 0.05 and ^##^*P* < 0.01 vs. hypoxia, ^§^*P* < 0.05 and ^§§^*P* < 0.01 vs. hypoxia+DHA, ^^^*P* < 0.05 vs. hypoxia+DHA+CQ, *n* = 3. (d) Double immunofluorescence of LC3 (red) and p62 (green) in NMCs (scale bar 10 *μ*m). (e and f) Corresponding quantitative analysis of LC3-II and p62 fluorescence intensity. Data are means ± SEM. ^∗∗^*P* < 0.01 vs. control, ^#^*P* < 0.05 vs. hypoxia, ^§§§^*P* < 0.01 vs. hypoxia+DHA, *n* = 3.

The western blot results indicated that expression of the autophagosome marker protein LC3-II in cardiomyocytes was significantly higher after 24 hours of hypoxia, while levels of the autophagy degradation marker p62 were lower. After DHA intervention, the expression of LC3-II proteins in cardiomyocytes further increased, while levels of p62 protein further decreased (Figures 4(a)–4(c)). These results imply that DHA can promote both production of autophagosomes and degradation of autophagy lysosomes in this myocardial hypoxia injury model.

To further confirm the role of DHA in the regulation of autophagy flux, CQ (an autophagy inhibitor) was used to block the renewal of autophagosomes for autophagy flux. The expression of the LC3-II and p62 proteins was significantly higher in the hypoxia+DHA+CQ and the hypoxia+CQ groups. These results indicate that CQ is involved in the inhibition of autophagosome degradation and blocks autophagy flow in myocardial hypoxia injury.

Further comparison revealed that expression of LC3-II protein was higher in the hypoxia+DHA+CQ group than in the hypoxia+CQ group (Figures 4(a)–4(c)). These results imply that DHA regulates autophagy in the hypoxic injury model of cardiomyocytes by promoting autophagic lysosomal degradation.

Consistent with the western blot data, double-labeled immunofluorescence assays showed a significant increase in the fluorescence aggregation of LC3-II with DHA intervention, while p62 was significantly reduced. With CQ intervention, the fluorescence aggregation of both LC3-II and p62 increased significantly (Figures 4(d)–4(f)). This indicated that DHA can promote the generation of autophagosomes after hypoxic injury and also promote the degradation of autophagolysosomes and enhance autophagic flux.

### 3.4. DHA-Enhanced Autophagic Flux in Post-AMI Mice

**Figure 5:**
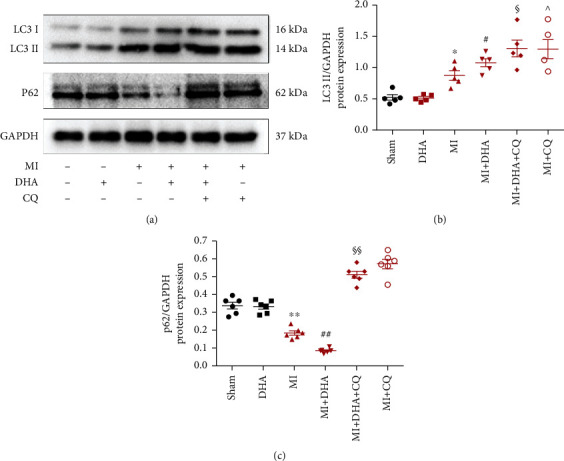
DHA-enhanced autophagic flux in post-AMI mice. (a) Western blotting analysis of LC3-II and p62 in mice. (b and c) Quantitative analysis of panel A. Data are means ± SEM. ^∗^*P* < 0.05 and ^∗∗^*P* < 0.01 vs. sham, ^#^*P* < 0.05 and ^##^*P* < 0.01 vs. MI, ^§^*P* < 0.05 and ^§§^*P* < 0.01 vs. MI+DHA, *n* = 6.

To test whether DHA enhances autophagy flux in post-AMI mice, expression levels of LC3 II and p62 were measured using western blot analysis. Compared to the MI group, expression of LC3 II was upregulated in the MI+DHA group, while expression of p62 was significantly reduced, indicating that DHA can effectively enhance autophagy flux in mice after AMI. After CQ treatment, the expression levels of both LC3 II and p62 increased significantly, confirming that CQ treatment can block renewal of autophagosomes. There was no significant difference in LC3 II or p62 expression between the MI+DHA+CQ and MI+CQ groups (Figure 5).

### 3.5. DHA-Enhanced Autophagic Flux Decreased Myocardial Apoptosis In Vitro and In Vivo

**Figure 6:**
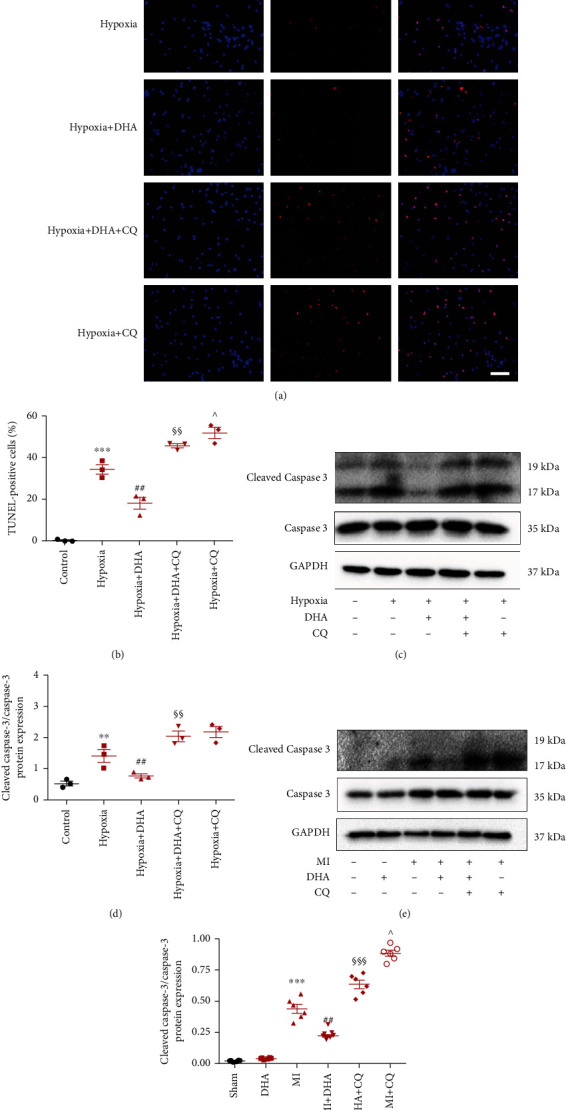
DHA-enhanced autophagic flux decreased myocardial apoptosis in vitro and in vivo. (a) Apoptosis of NMCs by TUNEL staining (red; original magnification ×200; scale bar 50 *μ*m). (b) Quantitative analysis of panel A. Data are means ± SEM. ^∗∗∗^*P* < 0.001 vs. control, ^##^*P* < 0.01 vs. hypoxia, ^§§^*P* < 0.01 vs. hypoxia+DHA, ^^^*P* < 0.05 vs. hypoxia+DHA+CQ, *n* = 3. (c and d) Western blots of LC3-II, p62, and GAPDH in hypoxia-treated NMCs. Data are means ± SEM. ^∗∗^*P* < 0.01 vs. control, ^##^*P* < 0.01 vs. hypoxia, ^§§^*P* < 0.01 vs. hypoxia+DHA, *n* = 3. (e and f) Western blots of LC3-II, p62, and GAPDH in post-MI mice. Data are means ± SEM. ^∗∗∗^*P* < 0.05 vs. sham, ^##^*P* < 0.05 vs. MI, ^§§§^*P* < 0.05 vs. MI+DHA, ^^^*P* < 0.05 vs. MI+DHA+CQ, *n* = 6.

In vitro TUNEL analysis showed that DHA treatment can significantly reduce NMC apoptosis after hypoxia exposure. The autophagy inhibitor CQ eliminated the antiapoptotic effects of DHA, which indicates that enhanced autophagy flux is necessary for the antiapoptotic effect of DHA (Figures 6(a) and 6(b)). The antiapoptotic characteristics of DHA were also observed with western blot analysis through the downregulation of cleaved caspase-3 and a lower cc3/c3 ratio. Following CQ stimulation in vitro and in vivo, this effect was reversed (Figures 6(c)–6(f)).

### 3.6. DHA-Enhanced Autophagic Flux Improved Cardiac Dysfunction

**Figure 7:**
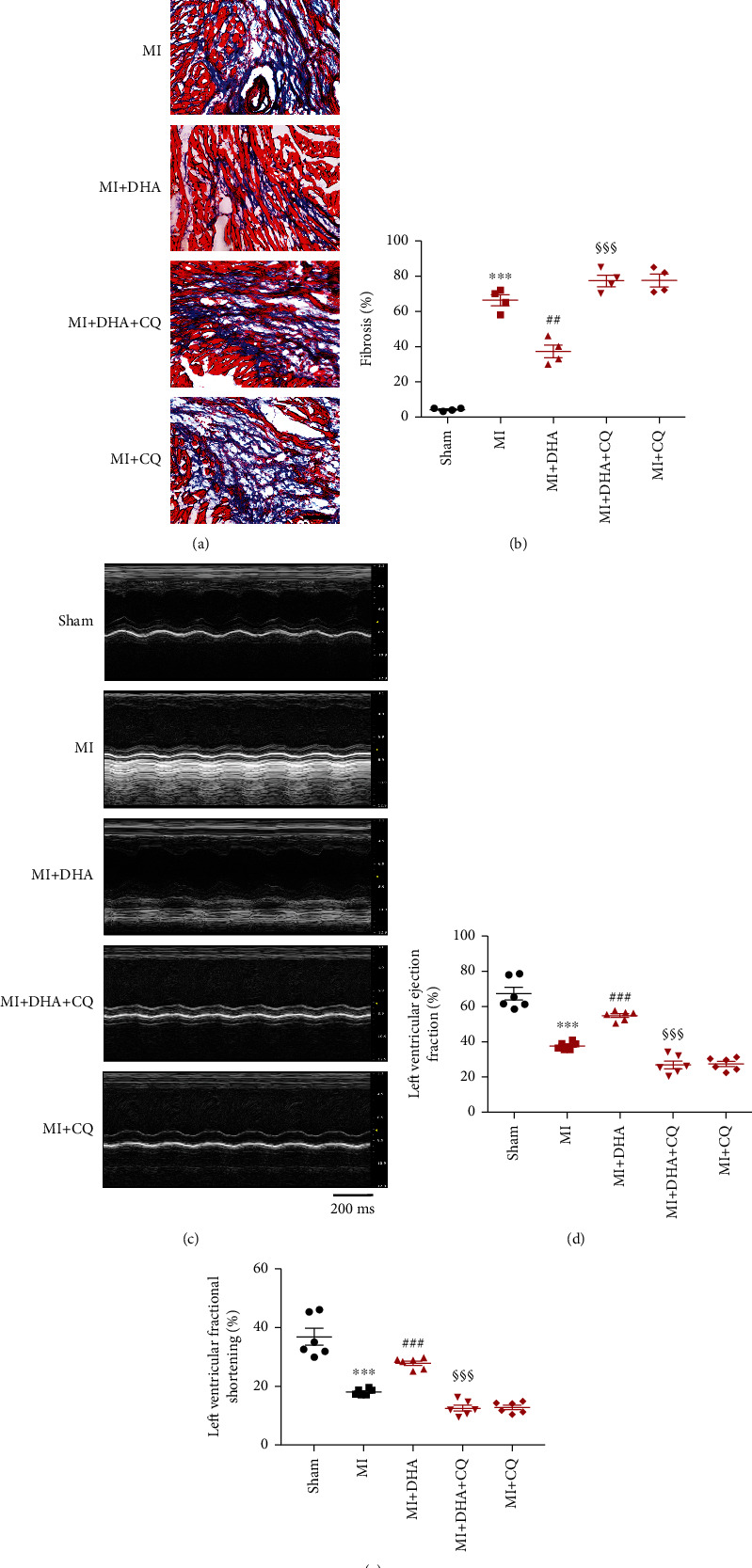
DHA-enhanced autophagic flux improved cardiac dysfunction. (a and b) Myocardial fibrosis assessed by Masson's staining (original magnification ×200; scale bar 50 *μ*m). (c and d) Heart function analysed by echocardiography. Data are means ± SEM. ^∗∗∗^*P* < 0.001 vs. sham, ^##^*P* < 0.01 and ^###^*P* < 0.001 vs. MI, ^§§^*P* < 0.01 and ^§§§^*P* < 0.001 vs. MI+DHA, *n* = 6.

We investigated whether DHA exerts a cardioprotective effect by enhancing autophagy flux. Masson's staining showed that DHA significantly reduced the myocardial fibrosis caused by AMI, but this effect was partially eliminated by CQ cotreatment (Figures 7(a) and 7(b)). M-mode echocardiography showed that DHA can improve LVFS and LVEF values, but CQ treatment partially prevented this effect; this indicates that autophagy flux is involved in the improvement of heart function induced by DHA (Figures 7(c)–7(e)). However, compared to the MI+DHA group, CQ did not significantly attenuate DHA-improved LVIDd and LVIDs values (Figure S2). These data indicate that DHA can reduce MI-related cardiac dysfunction by promoting autophagy flux.

### 3.7. DHA-Promoted Autophagic Flux via the AMPK/mTOR Pathway in Hypoxia-Treated NMCs

**Figure 8:**
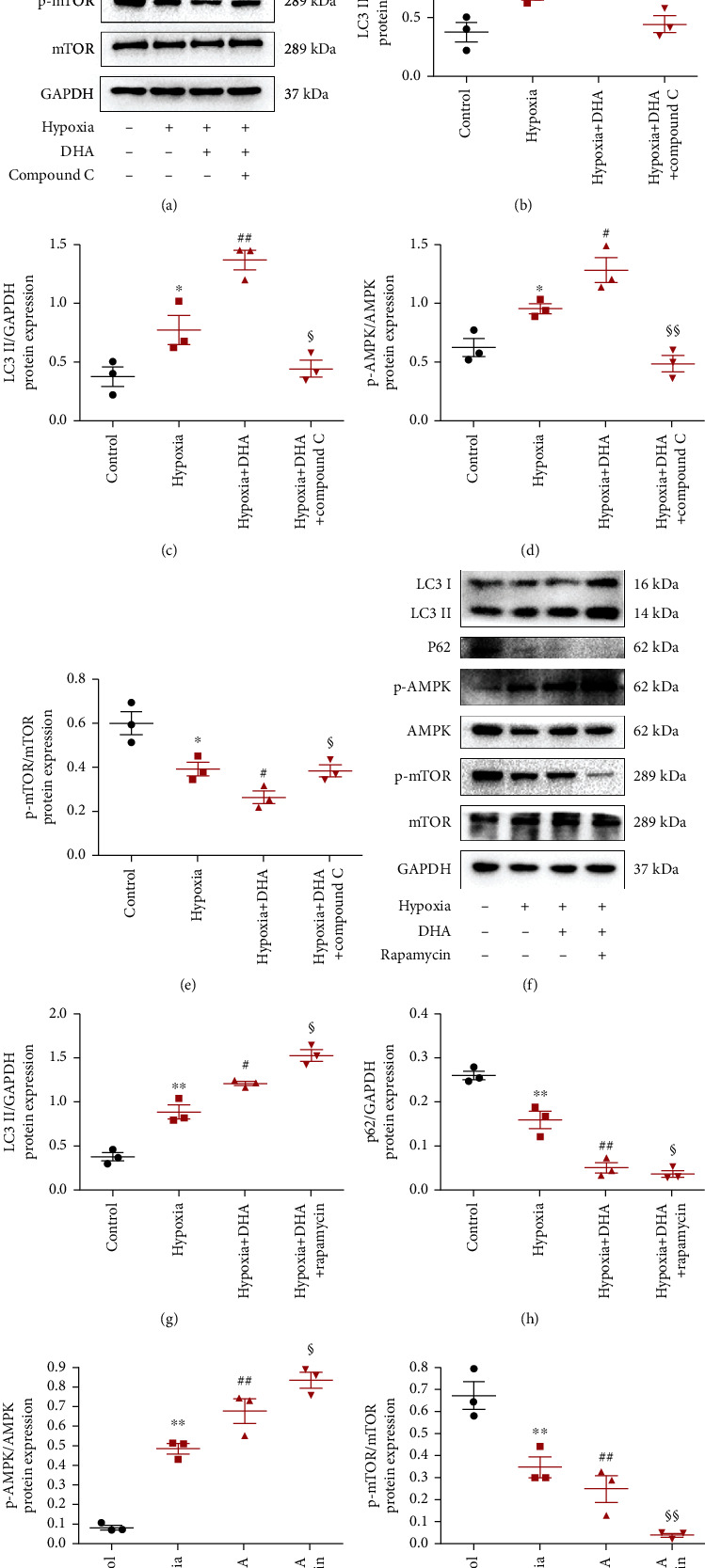
DHA promoted autophagic flux via the AMPK/mTOR pathway in hypoxia-treated NMCs. (a and f) P-AMPK, AMPK, p-mTOR, mTOR, LC3-II, and p62 protein levels in NMCs by western blotting. (b–e and g–j) Quantitative analysis of p-AMPK/AMPK, p-mTOR/mTOR, LC3-II/GAPDH, and p62/GAPDH levels. Data are means ± SEM. ^∗^*P* < 0.05 and ^∗∗^*P* < 0.01 vs. control, ^#^*P* < 0.05 and ^##^*P* < 0.01 vs. hypoxia, ^§^*P* < 0.05 and ^§§^*P* < 0.01 vs. hypoxia+DHA, *n* = 3.

Previous studies have shown that the AMPK/mTOR signaling pathway plays a crucial role in the activation of autophagic flux [15, 23, 24]. We hypothesized that this pathway is involved in activation of DHA's autophagic flux. Therefore, we used the AMPK-specific inhibitor compound C to study whether AMPK/mTOR signaling is essential for DHA-induced autophagic flux. DHA treatment significantly increased the phosphorylation of AMPK and decreased the phosphorylation of mTOR in vitro. Following treatment with compound C, the phosphorylation of AMPK was blocked and the inhibitory effect of DHA on mTOR phosphorylation was eliminated. Following compound C exposure, LC3-II decreased significantly, and p62 rose to a peak; this implies that in this myocardial hypoxia injury model, compound C's inhibitory effect on AMPK prevented activation of autophagy flux (Figures 8(a)–8(e)).

Rapamycin further induced phosphorylation of AMPK and inhibited phosphorylation of mTOR by DHA. Moreover, LC3-II was increased and p62 was decreased (Figures 8(f)–8(j)). This indicates that in hypoxic cardiomyocytes, DHA functions synergistically with rapamycin to inhibit mTOR phosphorylation and promote autophagy. These results prove that DHA enhances autophagy flux through the AMPK/mTOR pathway.

## 4. Discussion

This study demonstrated for the first time that DHA exerts a cardioprotective effect on AMI in mice by enhancing autophagy flux. Our results show that DHA can alleviate the myocardial damage and cardiac dysfunction caused by AMI; that DHA enhanced autophagy and thereby reduced apoptosis in the heart; and that DHA enhanced autophagy flux through AMPK/mTOR signaling in hypoxia-induced cardiomyocytes.

AMI is a serious cardiovascular disease that is often accompanied by major complications such as heart failure and arrhythmia. The development of treatment methods such as thrombolysis and PCI has increased the AMI survival rate. However, the death of myocardial cells caused by the initial stage of hypoxia leads to remodeling of the ventricular structure and impairs the pumping function of the heart during the recovery period, which eventually leads to heart failure. It has been suggested that omega-3 fatty acids have little effect on the mortality and adverse events of adult MI, but this may be related to a patient's previous underlying diseases [25]. On the other hand, it has also been suggested that eicosapentaenoic and DHA supplementation can prevent cardiovascular disease and that the protective effect is closely related to the dose [26]. DHA has previously been reported to play a protective role in ischemic diseases, so, what is the role and mechanism of DHA in AMI? In this study, CCK8 analysis and survival analysis were used to select and verify appropriate drug concentrations for follow-up studies. TTC staining, cardiac ultrasound, and Masson's staining were used in our study to confirm that in a mouse AMI model DHA pretreatment reduced the area of MI, improved heart function, reduced myocardial fibrosis, improved ventricular remodeling, and provided a cardioprotective effect. However, the mechanism by which DHA exerts myocardial protective effects is unknown.

Apoptosis, or type I programmed cell death, is characterized mainly by the formation of apoptotic bodies. Apoptosis is the major process responsible for myocardial cell death during MI [27]. Our research indicates that DHA pretreatment significantly reduces apoptosis-related proteins after MI. In recent years, a large number of studies have confirmed crosstalk between autophagy and apoptosis [28–30]. Indeed, autophagy can inhibit cell apoptosis and downregulation of autophagy can increase apoptosis [31]. However, it is not known whether the antiapoptotic effect of DHA after AMI is also regulated by autophagy.

Cardiovascular diseases and conditions, such as heart failure, myocardial hypertrophy, and myocardial ischemia damage can promote upregulation of autophagy. Hamacher et al. found that the level of autophagy increased during myocardial ischemia, reducing infarct size and improving cardiac function [32]. However, excessive autophagy can accelerate the degradation of functional organelles and important proteins and exacerbate cell death [33]. These contradictory findings may be explained by the level of autophagy flux.

Macroautophagy is the most widely studied autophagic process and involves four steps: autophagy precursor formation, autophagosome formation, autophagosome and lysosome fusion, and autophagic lysosome degradation (autophagy flow) [34]. The upregulation of autophagy may occur due to increased autophagosome formation or impaired degradation. When evaluating autophagy levels, autophagy flow should be included in the analysis. The patency of autophagosome degradation is generally evaluated using p62 and LC3-II. When the expression levels of both p62 and LC3-II increase, autophagy flow is inhibited. When the expression of p62 decreases and LC3-II expression increases, autophagy flow is enhanced. We applied the autophagic flow inhibitor CQ and found that it blocked the protective effects of DHA pretreatment. The results indicate that CQ blocks myocardial autophagic flow both in vivo and in vitro. DHA's antiapoptotic effect was significantly weakened, and “apoptosis executive protein” expression and cell apoptosis increased. Therefore, DHA can have an antiapoptotic effect in AMI by enhancing autophagy. However, the role of DHA in autophagy flow regulation is not yet fully understood.

We found that after MI or hypoxia, DHA increased LC3-II expression and decreased that of the autophagy marker p62. CQ significantly increased levels of both LC3-II and p62. Therefore, DHA may enhance autophagy flux by promoting autophagosome production, as well as autophagy-lysosomal degradation post-AMI. CQ reversed this effect. Further comparisons between the DHA+CQ and CQ-alone groups found no difference in LC3-II levels, indicating that DHA failed to promote autophagosome production after blocking the flow of autophagy. We speculate that DHA enhances autophagy flux in AMI and may primarily promote autophagolysosome degradation.

DHA activates AMPK in many diseases [16, 35]. The activation of AMPK can occur under various physiological and pathological states, such as exercise, ischemia and hypoxia, trauma, and inflammation [36–44]. In a mouse model of myocardial ischemia/reperfusion, melatonin regulated OPA1 by activating AMPK, thereby maintaining mitochondrial function and reducing myocardial cell death. While OPA1 is inhibited, the cardioprotective effect of melatonin is eliminated by AMPK blockers [40]. The results of an ischemia/reperfusion model of *α*2-AMPK knockout mice showed that *α* subunit-deficient mice had energy uptake disorders, which exacerbated ischemic injury and cardiac dysfunction [41]. Previous studies have shown that AMPK negatively regulates mTOR and participates in the regulation of autophagy [45–47]. However, the mechanism underlying the effect of DHA on autophagy is not fully understood.

Compound C, an APMK inhibitor, was used in our study to show that after hypoxia injury, DHA not only promoted AMPK activation but also inhibited mTOR phosphorylation and enhanced autophagy flux. However, following compound C treatment, the phosphorylation of myocardial AMPK was blocked, myocardial LC3-II levels decreased significantly, and p62 rose to a peak. Therefore, we speculate that the enhanced autophagy flux from DHA in hypoxic injury may be mediated by the AMPK/mTOR signaling pathway. However, the exact mechanism by which DHA activates AMPK signal transduction in post-AMI has not been fully studied and further exploration is needed.

**Figure 9:**
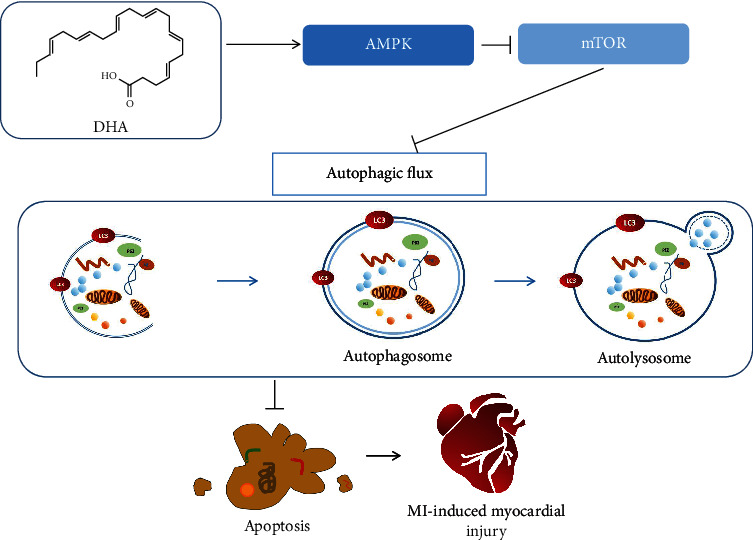
The DHA-mediated AMPK/mTOR pathway enhanced autophagic flux to alleviate myocardial injury after MI.

In conclusion, our research shows that DHA promotes autophagic flux by activating the AMPK/mTOR pathway, to alleviate myocardial damage and cardiac dysfunction after AMI. Pharmacological or nutritional intervention with DHA shows promise to prevent or treat AMI (Figure 9).

## Data Availability

The data used to support the findings of this study are available from the corresponding author upon request.

## Abbreviations

DHA: Docosahexaenoic acid
AMI: Acute myocardial infarction
NMCs: Neonatal mouse cardiomyocytes
CQ: Chloroquine
AMPK: Adenosine monophosphate-activated protein kinase
mTOR: Mammalian target of rapamycin
GAPDH: Glyceraldehyde 3-phosphate dehydrogenase
LC3: Microtubule-associated protein light chain 3
p62: Sequestosome 1
TTC: Triphenyltetrazolium chloride
LV: Left ventricle
LVIDd: Left ventricle internal diameters at diastole
LVIDs: Left ventricle internal diameters at systole
LVEF: Left ventricle ejection fraction
LVFS: Left ventricle fraction shortening
OCT: Oxytetracycline
TUNEL: Terminal deoxynucleotidyl transferase dUTP nick end labelling.

## Supplementary Materials

Supplementary Materials Figure S1: (A&B) LVIDd and LVIDs analysed by echocardiography. Data are means ± SEM. ^∗∗∗^*P* < 0.001 vs. sham, ^###^*P* < 0.001 vs. MI, *n* = 6. Figure S2: (A&B) LVIDd and LVIDs analysed by echocardiography. Data are means ± SEM. ^∗∗∗^*P* < 0.001 vs. sham, ^#^*P* < 0.05 and ^##^*P* < 0.01 vs. MI, ^§^*P* < 0.05 and ^§§^*P* < 0.01 vs. MI+DHA, *n* = 6.
