# Supplementary material for: Docosahexaenoic Acid-Enhanced Autophagic Flux Improves Cardiac Dysfunction after Myocardial Infarction by Targeting the AMPK/mTOR Signaling Pathway
Source: Oxid Med Cell Longev. 2022 Feb 27;2022:1509421. doi: 10.1155/2022/1509421 (PMC8898772; doi:10.1155/2022/1509421)
Supplement: Supplementary Materials — Figure S1: (A&B) LVIDd and LVIDs analysed by echocardiography. Data are means ± SEM. ∗∗∗P < 0.001 vs. sham, ###P < 0.001 vs. MI, n = 6. Figure S2: (A&B) LVIDd and LVIDs analysed by echocardiography. Data are means ± SEM. ∗∗∗P < 0.001 vs. sham, #P < 0.05 and ##P < 0.01 vs. MI, §P < 0.05 and §§P < 0.01 vs. MI+DHA, n = 6. [file 1509421.f1.doc]

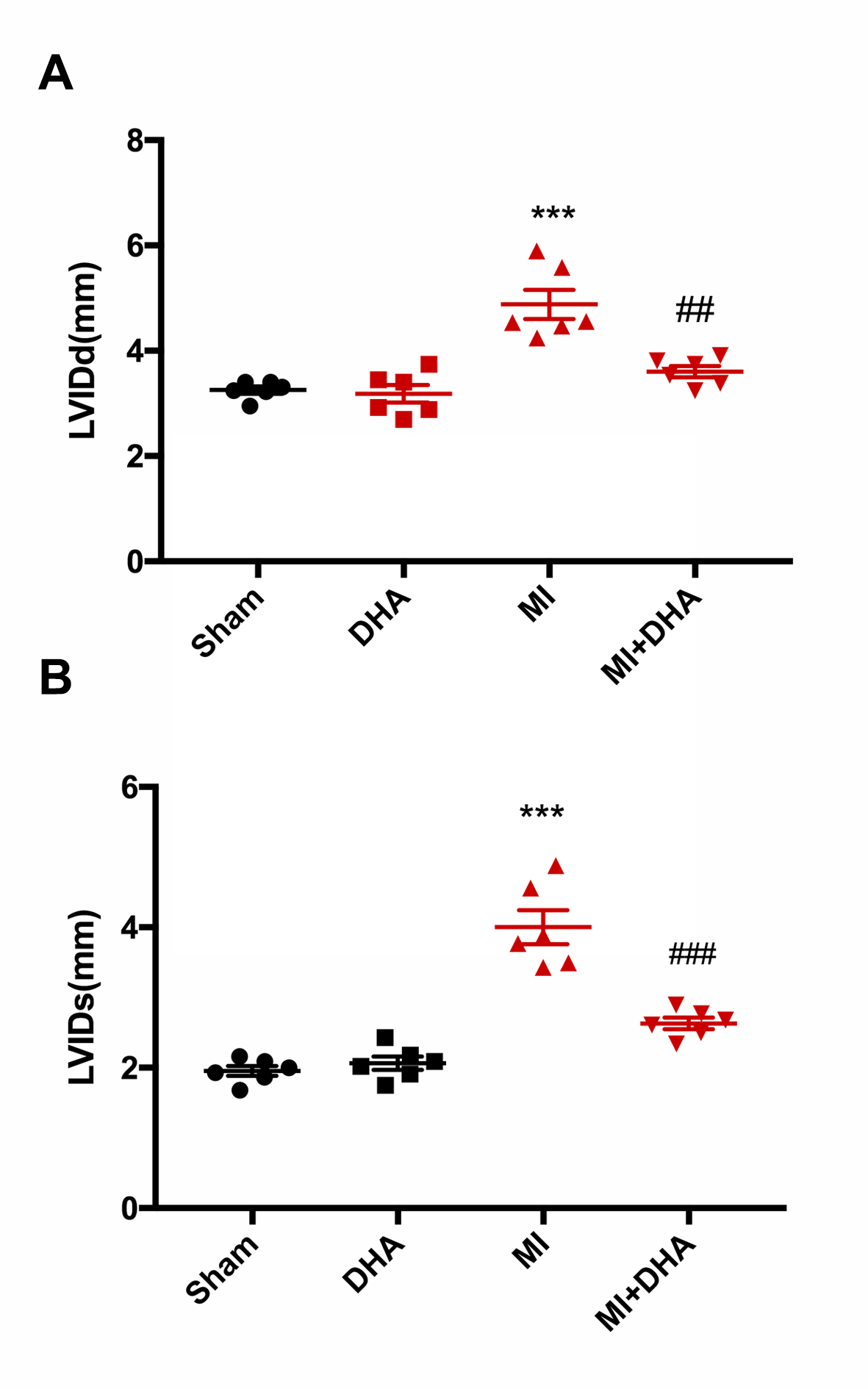


**Figure S1. (A&B) LVIDd and LVIDs analysed by echocardiography.** Data are means ± SEM. ^***^P < 0.001 vs. sham, ^###^P < 0.001 vs. MI, n = 6.


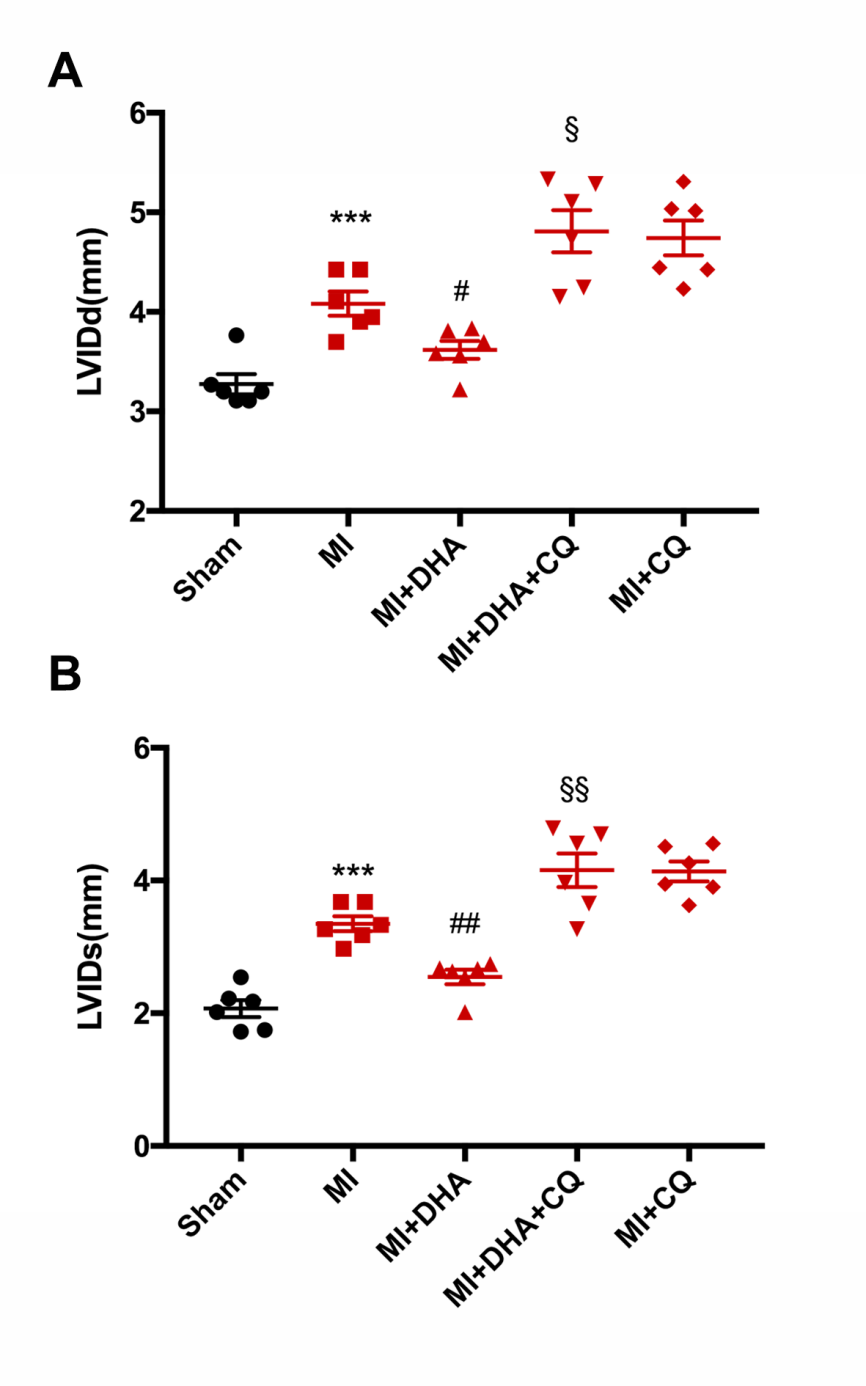


**Figure S2. (A&B) LVIDd and LVIDs analysed by echocardiography.** Data are means ± SEM. ^***^P < 0.001 vs. sham, ^#^P < 0.05 and ^##^P < 0.01 vs. MI, ^§^P < 0.05 and ^§§^P < 0.01 vs. MI + DHA, n = 6.
